# Expansion of the gamma-gliadin gene family in *Aegilops* and *Triticum*

**DOI:** 10.1186/1471-2148-12-215

**Published:** 2012-11-08

**Authors:** Svetlana V Goryunova, Elma MJ Salentijn, Nadejda N Chikida, Elena Z Kochieva, Ingrid M van der Meer, Luud JWJ Gilissen, Marinus JM Smulders

**Affiliations:** 1 Wageningen UR Plant Breeding, Wageningen UR, P.O. Box 16, Wageningen, NL-6700 AA, The Netherlands; 2 Vavilov Institute of General Genetics, Russian Academy of Sciences, Moscow, 119991, Russia; 3 Plant Research International, Wageningen UR, P.O. Box 16, Wageningen, NL-6700 AA, The Netherlands; 4 All-Russian Institute of Plant Industry, St. Petersburg, 190000, Russia; 5 Bioengineering Center, Moscow, 117312, Russia; 6 Allergy Consortium, Wageningen, The Netherlands

**Keywords:** Gamma-gliadin, Wheat, *Triticum*, *Aegilops*, Multigene family, Evolution

## Abstract

**Background:**

The gamma-gliadins are considered to be the oldest of the gliadin family of storage proteins in *Aegilops/Triticum*. However, the expansion of this multigene family has not been studied in an evolutionary perspective.

**Results:**

We have cloned 59 gamma-gliadin genes from *Aegilops* and *Triticum* species (*Aegilops caudata* L., *Aegilops comosa* Sm. in Sibth. & Sm., *Aegilops mutica* Boiss., *Aegilops speltoides* Tausch, *Aegilops tauschii* Coss., *Aegilops umbellulata* Zhuk., *Aegilops uniaristata* Vis., and *Triticum monococcum* L.) representing eight different genomes: A^m^, B/S, C, D, M, N, T and U. Overall, 15% of the sequences contained internal stop codons resulting in pseudogenes, but this percentage was variable among genomes, up to over 50% in *Ae. umbellulata*. The most common length of the deduced protein, including the signal peptide, was 302 amino acids, but the length varied from 215 to 362 amino acids, both obtained from *Ae. speltoides.* Most genes encoded proteins with eight cysteines. However, all *Aegilops* species had genes that encoded a gamma-gliadin protein of 302 amino acids with an additional cysteine. These conserved nine-cysteine gamma-gliadins may perform a specific function, possibly as chain terminators in gluten network formation in protein bodies during endosperm development. A phylogenetic analysis of gamma-gliadins derived from *Aegilops* and *Triticum* species and the related genera *Lophopyrum*, *Crithopsis*, and *Dasypyrum* showed six groups of genes. Most *Aegilops* species contained gamma-gliadin genes from several of these groups, which also included sequences from the genera *Lophopyrum, Crithopsis,* and *Dasypyrum.* Hordein and secalin sequences formed separate groups.

**Conclusions:**

We present a model for the evolution of the gamma-gliadins from which we deduce that the most recent common ancestor (MRCA) of *Aegilops/Triticum-Dasypyrum-Lophopyrum-Crithopsis* already had four groups of gamma-gliadin sequences, presumably the result of two rounds of duplication of the locus.

## Background

Prolamin storage proteins are produced in large amounts in the developing endosperm of Triticeae species. These storage proteins are a complex mixture of alpha/beta-, gamma- and omega-gliadins and high- and low molecular weight glutenins, collectively called ‘gluten’ in wheat. They are encoded by medium to large multigene families. For example, the alpha-gliadins are encoded by a complex gene family with estimates for copy number that range from 25–35 copies [1] to 100 [2] or even 150 copies [3] per haploid genome, most of which (72-95%) are pseudogenes [3,4]. Sequence similarity of alpha-gliadins from bread wheat to alpha-gliadins from diploid *Aegilops/Triticum* species, which are close relatives of the diploid ancestors of bread wheat, demonstrated that there are three distinct groups of alpha-gliadins, one for each of the three homoeologous loci in hexaploid bread wheat [4]. This is consistent with the notion that the expansion of this gene family took place after the ancestors of the different genomes of *Aegilops/Triticum* became separated.

The gamma-gliadins are considered to be the most ancient of the gliadins and LMW-glutenins [5]. In bread wheat they are encoded by the homoeologous *Gli-1* loci (*Gli-A1*, *Gli-B1* and *Gli-D1*), located on the short arms of the homoeologous chromosomes 1 [6,7]. In the variety Chinese Spring the number of gamma-gliadins was preliminary estimated at 15–40 [8,9] and, in contrast to the situation in alpha-gliadins, only a small fraction (~14%) of the gamma-gliadin genes in hexaploid bread wheat consisted of pseudogenes [10]. Nevertheless, sequence analysis showed that the gamma-gliadins form a highly diverse gene family [9,10].

The large majority of the gamma-gliadin sequences available in Genbank are from tetraploid *Triticum durum* (A and B genomes) and hexaploid *Triticum aestivum* (A, B and D genomes), diploid *Triticum monococcum* (A genome) and diploid *Aegilops* species with S and D genomes (the B genome is closely related to the S genome of *Aegilops speltoides*, [11,12]). Using such a collection of gamma-gliadin sequences Qi et al. [10] classified gamma-gliadins into 17 subgroups, most of which had 8 cysteine residues per protein, but 7, 8, and 10 residues also occurred. The cysteine residues form sulphur bridges, and proteins with unequal numbers of cysteins can covalently bind to a network of HMW glutenins and other gluten proteins [13]. Of these 17 subgroups those with A genome gamma-gliadins appeared to be distinct from the subgroups that contain B (S) and/or D genome genes. As only these three diploid progenitor genomes were included, the study did not provide insight in the evolutionary history of the gamma-gliadins. Wang et al. [14] recognised four groups of gamma-gliadins.

Although wheat storage proteins form multigene families, their phylogeny can be established effectively using knowledge on the phylogenetic and evolutionary relationships among *Triticum* and *Aegilops* genomes. Zhang et al. [15] and Li et al. [16] studied the HMW glutenin subunits, whereas Zhang et al. [17] and Wang et al. [18] focused on LMW glutenin subunits. From this it appears that, in case of multigene families, it may be necessary to infer relationships at the level of groups of closely related genes rather than for individual genes.

Here we have studied the evolution of gamma-gliadins. For this we have complemented the available gamma-gliadin sequences from diploid *Aegilops*/*Triticum* species with novel sequences from diploid species representing the other main genome types in *Aegilops/Triticum*: the C, M, N, U, and T genomes. Our analysis of these genes shows that there are six groups of gamma-gliadins that occur in different combinations across all the genomes. We present a model for gene duplications and losses that is consistent with our data. Our model indicates that at least some gene duplications are presumed to predate the most recent common ancestor (MRCA) of all *Aegilops/Triticum* genomes.

## Methods

### Plant material

In this paper we followed the classification of Van Slageren [19] with the exception of *Ae. mutica*, that was regarded by Van Slageren as a separate genus, *Ambylopyrum* (Jaub. & Spach) Eig. We used accessions of 7 diploid Aegilops species: *Aegilops caudata* L. (к-2255, Turkey, C genome), *Aegilops tauschii* Coss. (к-1368, Uzbekistan, D), *Aegilops comosa* Sm. in Sibth. & Sm. (к-2272, Asia Minor, M), *Aegilops uniaristata* Vis. (к-650, Greece, N), *Aegilops speltoides* Tausch (CGN10682 and CGN10684, S), *Aegilops mutica* Boiss. (к-1581, Turkey, T), and *Aegilops umbellulata* Zhuk. (к-1588, Afghanistan, U), as well as *Triticum monococcum* L. (CGN10542, A). The accessions starting with “к” were obtained from the All-Russian Institute of Plant Industry (St. Petersburg, Russia). CGN numbers are from the Centre for Genetic Resources (Wageningen, The Netherlands). The set of species represent all main genome types in *Aegilops/Triticum*. Three of the species analysed have genomes closely related to genomes of cultivated wheat *T. durum* (AB genome) and *T. aestivum* (ABD genome): *Ae. speltoides, Ae. tauschii, and T. monococcum*.

### Cloning and sequencing

DNA was isolated from young fresh leaves using the Edwards procedure modified by Dorokhov and Klocke [20,21]. The primers used for amplification of gamma-gliadin sequences were complementary to 3’ and 5’ conserved regions of gamma-gliadins. The forward primer γ1F: 5’-atgaagaccttactcatcc-3’ resides in the signal peptide, the reverse primer γ11R: 5'-ggacaWagacRttgcacatg-3' in domain V. The PCR cycling conditions: 5 min. at 94°C followed by 24 cycles (94°C for 1 min., 53°C for 1 min, 72°C for 2 min), 72°C for 10 min, in 25 μl reaction volume. The PCR products were cloned into the pCRII-TOPO vector (Invitrogen) and sequenced using the M13 forward (5’-cgccagggttttcccagtcacgac-3’) and reverse primer (5’-agcggataacaatttcacacagga-3’) and two additional internal primers γFi2: 5’-ccc(ac)tgcaagaat(at)t(ct)c-3’ and γRi2: 5’-g(ag)a(at)attcttgca(gt)ggg-3’. This produced four overlapping reads for each clone.

### Sequence analysis

The reads were merged per clone and the sequence data were manually checked using SeqMan (DNASTAR) to exclude sequencing mistakes. Sequences that were suspected to be chimeric, that lacked 5’ or 3’ ends, or that had a very long deletion (sequence length in the alignment less than 600 bp) were excluded from the phylogenetic analysis. Each PCR product was a mixture of sequences from different genes, so many of the 11–81 clones obtained from one PCR reaction were independent. However, some duplicate clones may be derived from the same gene, possibly even from the same amplification product with a particular PCR error. Therefore all remaining 335 sequences were conservatively organized into 59 contigs (sets of overlapping DNA sequences) with 99% similarity. The consensus sequences of the contigs thus obtained were used for further statistic/phylogenetic analysis. One to three sequences representing each consensus sequence were submitted to Genbank. In total 69 novel gamma-gliadin sequences were submitted, representing 59 contig consensus sequences. The length of the partial gamma-gliadin sequences obtained varied from 545 to 986 base pairs and corresponded to a part of full-length open reading frame region of gamma-gliadins which is 648–1089 bp in length. They encode gamma-gliadins of 215–362 amino acids. These sequences are probably not the complete set of gamma-gliadin genes from each of the accessions, but the aim was to clone a sufficient number of genes from each accession to obtain representatives of all distinct groups of gamma-gliadins for a phylogenetic analysis, rather than a complete set of gamma-gliadin genes and pseudogenes from all accessions.

For the phylogenetic analysis the genes cloned and sequenced here were supplemented with sequences of diploid *Triticum* and *Aegilops* species and of the related genera *Lophopyrum*, *Crithiopsis*, and *Dasypyrum* from EMBL/Genbank (as present in August 2011). These were organized in the same way in contigs of 99% sequence similarity; a total of 145 sequences and 68 contigs (Table 1). All 127 contigs (59 composed of novel sequences and 68 of EMBL/Genbank-derived sequences) were trimmed to represent the same part of the gene. One gamma-hordein sequence (AY338365 from *Hordeum chilense*) and three secalins (EU368041 from *Secale cereale*, EF432546 from *Secale sylvestre*, and HQ266670 from *Secale strictum*) were included as outgroups, as the sequence alignment already indicated that they are more distant.

**Table 1:** The 127 gamma-gliadin sequences analysed in this study

**Species**	**Accession**	**Genome**	**N seq**	**N contigs (99%)**	**Genes**	**Pseudo-genes**	**Length (bp)**	**Genbank accession nr**
**Cloned in this study**								
*T. monococcum*	CGN10542	Am	23	5	4	1	759-939	JQ269804-JQ269808
*Ae. caudata*	к2255	C	50	12	9	3	909-948	JQ269703-JQ269716
*Ae. comosa*	к2272	M	32	5	3	2	887-909	JQ269717-JQ269721
*Ae. uniaristata*	к650	N	35	7	6	1	873-924	JQ269742-JQ269750
*Ae. mutica*	к1581	T	35	8	7	1	882-909	JQ269722-JQ269729
*Ae. tauschii*	к1368	D	32	4	4	0	879-897	JQ269789-JQ269792
*Ae. umbellulata*	к1588	U	36	10	5	5	873-928	JQ269730-JQ269741
*Ae. speltoides*	CGN10682	S	11	3	3	0	648, 909	JQ269774-JQ269778
*Ae. speltoides*	CGN10684	S	81	5	5	0	873-1089	JQ269751-JQ269757
**Total cloned in this study**			335	59	46	13		
**Already present in Genbank/EMBL/DDBJ (in August 2011)**								
*Ae. searsii*			9	3	3	0		
*Ae. bicornis*			13	3	3	0		
*Ae. longissima*			10	4	3	1		
*Ae. sharonensis*			8	5	4	1		
*Ae. speltoides*			11	3	3	0		
*Ae. tauschii*			10	4	4	0		
*T. monococcum*			30	14	13	1		
*T. urartu*			14	5	4	1		
*Crithopsis delileana*			2	2	2	0		
*Lophopyrum elongatum*			16	14	8	6		
*Dasypyrum sp.*			22	11	5	6		
**Total in Genbank/EMBL/DDBJ**			145	68	52	16		

Both the nucleotide and the deduced amino acid sequences of the gamma-gliadin dataset were aligned using MEGA4 [22], and Maximum-Likelihood (ML) analysis was performed with PhyML 3.0 ( http://www.phylogeny.fr[23,24]) using the GTR-substitution model for nucleotide data and WAG-model for amino acid data. SH-like approximate likelihood-ratio test was used for estimation of branch support [25]. MEGA4 used the complete alignment, while the ML-program at PhyML excluded all sites with deletions. When we used the pairwise deletion option for neighbour joining (NJ) in MEGA4 we obtained the same tree topology.

The number of base differences per site, number of synonymous differences per synonymous site and number of non-synonymous differences per non-synonymous site from averaging over all sequence pairs within each group and overall sequences was calculated using the method of Nei and Gojobori [26] with incorporation of the Jukes-Cantor correction in MEGA4. Standard error estimates were obtained by a bootstrap procedure (1000 replicates). All positions containing alignment gaps and missing data were eliminated only in pairwise sequence comparisons (Pairwise deletion option). The ratio between synonymous substitutions per site (*d*_S_) and non-synonymous substitutions per site (*d*_N_) and (*d*_S_ /*d*_N_ ratio) was calculated.

To study the selection pressure on gamma-gliadin sequences the codon-based test for selection (Z-test) was performed for sequences of each of groups and for overall dataset. The variance was computed using bootstrapping (1000 replicates). To analyse differences in selection pressure on full open reading frame (ORF) and pseudogene gamma-gliadin sequences the number of synonymous (Ks) and non-synonymous substitutions (Ka) per site were calculated from pairwise comparisons for ORF and pseudogene sequence pairs using the method of Nei and Gojobori [26]. The values obtained were used for a scatter plot in Excel.

## Results

### Gamma-gliadin sequences

In order to analyse genetic diversity and the evolution of the gamma-gliadin multigene family 335 gamma-gliadin sequences were cloned and sequenced from species representing all main genome types in *Aegilops/Triticum* (A, B/S, D, G, M, N, U, and T genomes). The aim was to clone and sequence a sufficient number of genes from each accession to obtain representatives of all distinct groups. The sequences were assembled into contigs at 99% homology at nucleotide level (Additional file 1). The contigs with intact open reading frames represented 46 different predicted gamma-gliadin proteins (Table 1). Thirteen contigs (49 sequences) contained internal stop-codon or frame-shift mutations and were therefore considered to represent pseudogenes. The fraction pseudogene sequences differed among the eight *Aegilops/Triticum* species analysed. For example, more than half of all sequences of *Ae. umbellulata* were pseudogenes (20 of 35 sequences in 5 of 10 contigs), while no pseudogene contigs were present among 32 sequences from *Ae. tauschii* (Table 1).

**Figure 1:**
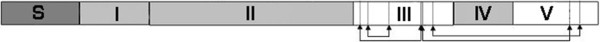
**Schematic overview of the structure of gamma-gliadins.** The proteins consist of a short N-terminal signal peptide (S) followed by a unique N-terminal domain (I) and a repetitive domain (II). Domain III contains most (often 6) of the cysteines. IV is rich in glutamine. Two conserved cysteines are in V. Eight cysteine residues (indicated with vertical lines) can form four interchain disulfide bonds (indicated as connections between lines). Figure after [9].

Figure 1 presents a schematic overview of the structure of gamma-gliadins, after [9] and [27]. The sequences of the predicted intact proteins varied in length considerably due to variation in the length of the repetitive domain (II) and the length of the glutamin-rich domain (IV). Most of the sequence length variation was observed among *Ae. speltoides* sequences, and both the shortest and the longest sequences were isolated from *Ae. speltoides*. 

### Clustering and phylogenetic analysis

**Figure 2:**
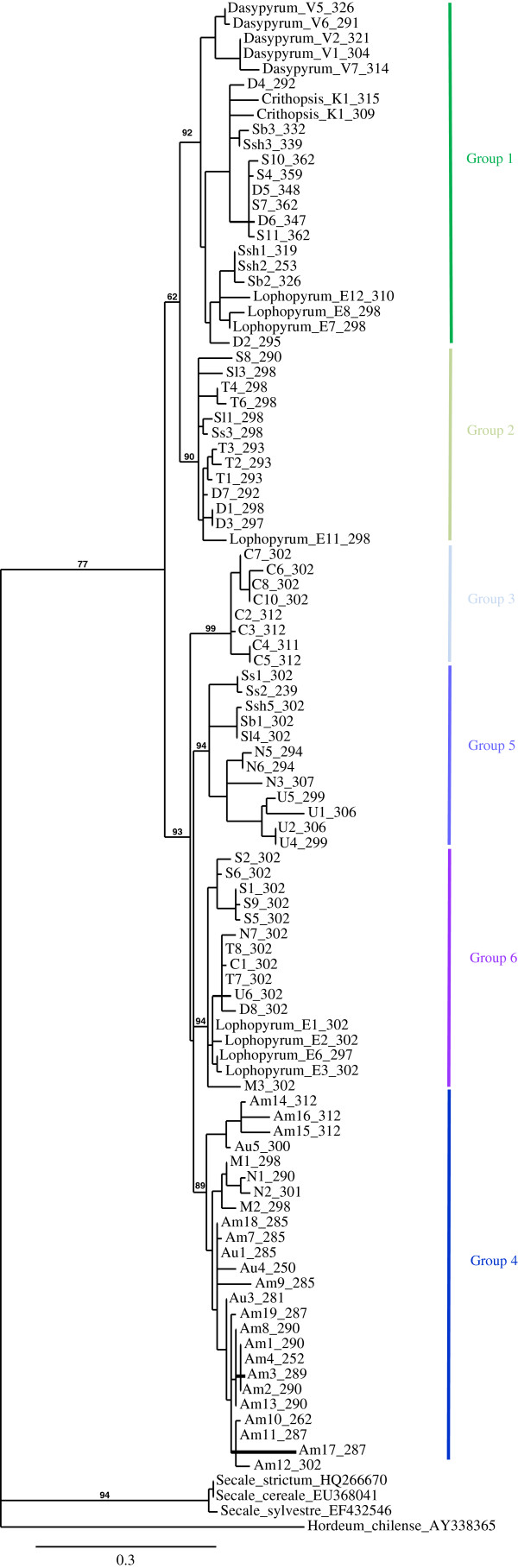
**Maximum-Likelihood (ML) tree of the gamma-gliadins (based on amino acid sequences).** A maximum-likelihood (ML) analysis was performed with PhyML 3.0. SH-like approximate likelihood-ratio test was used for estimation of branch support. Proteins with a length in the alignment less than 200 amino acids were excluded from the analysis. The gamma-gliadins fall into six groups (1–6 on the right) in two branches (1–2 and 3-4-5-6). Key for the sequence codes in Additional file 1.

An analysis of the sequences with a gamma-hordein as outgroup, resulted in a multiple sequence alignment (Additional file 2 contains the nucleotide alignment, Additional file 3 contains the amino acid alignment, both in Nexus format). The maximum-likelihood (ML) tree produced on the basis of the alignment contained a separate cluster of secalins and two well-supported groups of gliadins of unequal size: 53 consensus sequences belonged to the first group and 74 belonged to the second group (Additional file 4 contains the tree based on nucleotide sequences, Figure 2 shows the tree based on deduced amino acid sequences). In total six significant (bootstrap support value 84% or higher) groups were observed, two within the first branch (designated group 1 and 2) and four within the second branch (designated group 3–6). The groups contain sequences cloned here as well as sequences obtained from Genbank, and Genbank sequences do not form additional groups, indicating that we have cloned and sequenced sufficiently deep.

**Figure 3:**
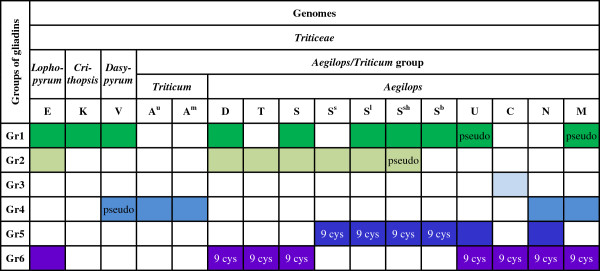
**Occurrence and absence of genes from different ancestral groups across the Aegilops/Triticum genomes.** Overview of the occurrence of genes from the six groups recognised from the maximum likelihood trees (Figure 2, Additional file 4), represented here as Gr1-Gr6 to the left, in all taxa for which we have gamma-gliadin sequences, sorted by genome (at the top). Color in a cell means present, empty means absent. Text in cells indicates additional features: ‘pseudo’ means that all sequences represent pseudogenes (i.e., with stopcodons); ‘9 cys’ indicates that all genes contain exactly 9 cysteines (all other gamma-gliadins generally contain 8 cysteines).

Sequences of *Ae. umbellulata* (U), *Ae. comosa* (M), *Ae. mutica* (T), *Ae. tauschii* (D), all species with an S genome (*Ae. speltoides* (S), *Ae. searsii* (S^s^), *Ae. bicornis* (S^b^), *Ae. sharonensis* (S^sh^) *and Ae. longissima* (S^l^)) occurred in both branches and in at least two unrelated groups (Figure 3). Sequences originating from *Triticum* species with an A genome (*T. monococcum* (A^m^) and *T. urartu* (A^u^), and *Aegilops* species *Ae. caudata* (C) and *Ae. uniaristata* (N) were restricted to the second branch. Within this second branch, all gamma-gliadin sequences from *T. monococcum* (*A*^*m*^) and *T. urartu* (*A*^*u*^) clustered in group 4. Group 3 consisted only of *Ae. caudata* (C) sequences, and it included all of them except one that was present in group 6. All groups except the *Ae. caudata*-specific group 3 included a mixture of sequences of three to seven species of *Aegilops/Triticum*. Each of the groups included terminal branches that are mainly species/genome-specific.

The gliadin sequences of *Dasypyrum, Lophopyrum* and *Crithopsis* included in the analysis were also positioned within the two branches despite the fact that *Triticum* and *Aegilops* are much more closely related and treated as one large genus by some authors [28,29]. The sequences of *Lophopyrum* clustered in groups 2 and 6, sequences of *Dasypyrum* clustered in groups 1 and 4 (in group 4 only pseudogenes, visible in the nucleotide maximum likelihood (ML) tree in Additional file 4), and those from *Crithopsis* clustered in group 1. Only groups 3 and 5 contained exclusively sequences of *Aegilops/Triticum* species.

### Genetic variation within and among the groups

The most polymorphic sequences were found in group 1. This group of sequences varied in length from 762 to 1089 bp, which means that it includes many of the shortest and all of the longest variants of the whole study. They were highly polymorphic with a codon-based evolutionary divergence (d) of 0.089 ± 0.005 (ds=0.191, dn=0.065) (Table 2, Additional file 5). Genes of this group are only maintained in the D and various S genomes and in the genera *Lophopyrum*, *Crithiopsis*, *Dasypyrum*. They occur as pseudogenes in the U and M genome (Figure 3). It thus appears that group 1 has undergone intensive diversification and death processes in most of the species analysed.

**Table 2:** Estimates of average evolutionary divergence over sequence pairs within groups

	**Pairwise Deletion**						
	**Synonymous mutations only**		**Non-synonymous mutations only**		**All substitutions**		
**Groups**	**dS**	**S.E.**	**dN**	**S.E.**	**d**	**S.E.**	**dN/dS**
**Gr 1**	0.191	0.019	0.065	0.006	0.089	0.005	0.340
**Gr 2**	0.092	0.014	0.03	0.004	0.042	0.004	0.326
**Gr 3**	0.072	0.012	0.034	0.005	0.044	0.005	0.472
**Gr 4**	0.177	0.019	0.048	0.006	0.074	0.005	0.271
**Gr 5**	0.111	0.016	0.043	0.005	0.059	0.004	0.387
**Gr 6**	0.087	0.012	0.029	0.004	0.041	0.004	0.333
**Gr 1 2**	0.198	0.019	0.066	0.005	0.09	0.006	0.333
**Gr 3 4 5 6**	0.187	0.017	0.065	0.006	0.09	0.005	0.348
**overall**	0.249	0.019	0.085	0.07	0.115	0.006	0.341

The least polymorphic are the group 6 gamma-gliadins. They are present in seven *Aegilops* genome types (T, D, U, C, N, M and S (only *Ae. speltoides*)) and in *Lophopyrum*. The *Aegilops* sequences of this group all have the same deduced ORF length of 909 bp, coding for a 302 amino acids gliadin protein. The average codon-based evolutionary divergence over sequence pairs within this group (d) is 0.041 ± 0.004 (ds=0.087, dn=0.029), which is only half of the group 1 gliadins. Interestingly, all *Aegilops* sequences of group 6 have an additional cysteine residue whereas in *Lophopyrum* sequences of group 6 the additional cysteine is not present, and here the predicted length of the protein is not 302 amino acids either. The cysteine can easily be formed by a single nucleotide change (TCC to TGC).

The *Aegilops* species that do not have group 6 gliadins are the S genome species except Ae. speltoides (S^s^, S^b^, S^sh^, S^l^ genomes), all of which have group 5 gliadins (Figure 3). These gliadins, although distinct in sequence composition, have the same length of 302 amino acids as the group 6 gliadins and have also an additional cysteine in the same position (except FJ006687, which has a large deletion). As a consequence, each *Aegilops* species contains a group of 9-cysteine gliadins, either from group 6 or from group 5. The U and N genomes contain group 6 sequences and group 5 sequences but, in contrast to group 5 sequences from S-genome *Aegilops* species, the U and N sequences from group 5 all contain only eight cysteins and are variable in length.

### Selection

The codon-based test for selection (Z-test) showed evidence for purifying selection in each of the six groups of sequences and also overall (Table 2). The ratio between synonymous and non-synonymous substitutions per site (*d*_S_/*d*_N_) for pairwise comparisons of sequences showed a relative excess of synonymous substitutions compared to non-synonymous substitutions in full open reading frame genes compared to genes with stop codons (pseudogenes) (see the trend line in Additional file 5). The difference in the ratios is comparable to those obtained for intact and pseudogene alpha-gliadins [4] but some of the values for dS as well as dN are higher, indicating that gamma-gliadins are an evolutionary older family.

## Discussion

The main genomes within the *Aegilops/Triticum* group (A, S/B, C, D, M, N, T, U) have split within an evolutionary short period, 2.5 to 4.5 MYA [30]. Multi-gene families have expanded in the same period as these genomes split. Here we obtained 59 new gamma-gliadin genes from eight genomes, and have analysed these data together with gene sequences in Genbank in the frame of gains and losses of groups of gamma-gliadin genes during the evolution of these species. This has produced new insight in how this multigene family has developed. Among the diversity of genes some groups show a remarkable stability of protein length and number of cysteines, suggesting functional relevance.

### A model for the evolution of gamma-gliadins

Evolution of multigene families occurs by duplication of gene clusters [31,32]. Gao et al. [33] showed evidence for multiple rounds of segmental duplication of omega-gliadin genes in wheat. The evolution of the gamma-gliadins appears to fit to the birth-and-death evolutionary model [34]. The sequence data obtained here allowed us to distinguish six groups of closely related gamma-gliadins (Figures 2 and 3, Additional file 4), which appear to be organised in two branches. These two ancestral branches predate the MRCA of the *Aegilops/Triticum* clade, as they also include sequences from the genera *Lophopyrum, Crithopsis,* and *Dasypyrum.* A hordein sequence from *Hordeum* and the secalins from *Secale* clustered outside the two main branches. A recent phylogenetic study of the Triticeae based on one chloroplastic and 26 nuclear gene sequences [35] placed *Secale* closer to *Aegilops* and *Triticum* than *Dasypyrum*, but also noted that the clade grouping these genera had evolved in a reticulated manner, and that their relationships are better represented by a multigenic network.

**Figure 4:**
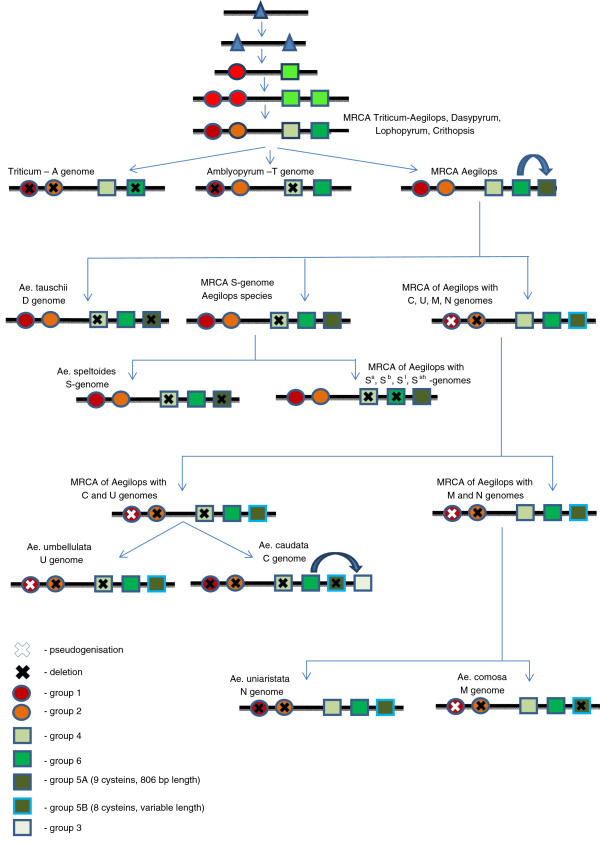
**Model for the evolution of groups of gamma-gliadins in*Aegilops/Triticum.*** The six groups proposed are based on the ML tree (Figure 2, Additional file 4) and occur in genomes as summarised in Figure 3. Note that in this model the order of the groups on the chromosome is arbitrary, and duplications of genes within each group are ignored. The occurrence of pseudogenes is only indicated when it affected complete groups, but some pseudogenes may occur in all groups. Note that each genome has either group 6 gliadins or group 5 gliadins with nine cysteines and constant length.

Based on a careful examination of the presence and absence of the six groups of gamma-gliadins we present a model for the evolution of this multigene family during the evolution of the *Aegilops/Triticum* (Figure 4). Note that in this model the order of the groups along the chromosome is arbitrary, and that repetitive DNA and non-gamma-gliadin genes that are present between gamma-gliadins [33] have been omitted. While developing this model we have assumed that our set of sequences (both cloned here and obtained from Genbank) is sufficiently deep to not have missed particular groups. Evidence supporting this notion is that (i) our sequences, obtained using PCR primers designed by us, fall into the same six groups as those of other diploid taxa from Genbank; (ii) all groups except the *Ae. caudata*-specific group 3 included a mixture of sequences of three to seven species of *Aegilops/Triticum*; (iii) the number of genes from one genome was not correlated with the number of groups into which they clustered. All *Ae. caudata* genes but one ended up in group 3, but we had cloned 12 different genes. *T. monococcum* genes ended up only in the lower branch, but we had as many as 19 different genes (Table 1). Finally, (iv) four of these groups were also recognised by other studies. One of the two groups missed by Wang et al. [14] was the *Ae. caudata*-specific group 3. 

### Gamma-gliadin duplication, pseudogenisation, and loss during *Aegilops*/*Triticum* genome evolution

The six groups of gamma-gliadins fall into two branches: one including group 1 and group 2 genes, and one including groups 3 to 6. In our evolutionary model the MRCA of the *Aegilops*/*Triticum* spp. already has four distinct groups of differentiated gamma-gliadin sequences, i.e., two from each branch (group 1, 2, 4 and 6, Figure 4). Almost all extant *Aegilops*/*Triticum* genomes include several distinct groups of gamma-gliadins. The only exception is the A genome of *Triticum*, which contains only group 4 gliadins. Consequently, its position in the model is the least supported, as loss of the other groups may have occurred at several points in time. The T genome lost group 4 and group 1 gliadins. A major split is between the D genome and the S genomes, that have lost the group 4 gliadins but maintained group 1 plus group 2 gliadins, and the genomes that lost group 1 and group 2 gliadins (M, N, U, C genomes). It is likely that these lineages have split from the MRCA of the other Aegilops genomes very early. This is consistent with taxonomic studies. *T. monococcum* and *T. urartu,* carrying two different modifications of the A genome, are usually treated together with polyploids carrying the A genome as a separate genus, *Triticum*[19,36-40]. *Ae. mutica* (T) appears to represent a separate evolutionary line within *Aegilops/Triticum* as this species shows many primitive characters. In some classifications it is treated as a separate genus, *Ambylopyrum*[19,41], or placed within a separate monotypic subgenus, *Ambylopyrum,* within *Aegilops*[39]. Cytogenetic studies [42] confirmed this isolated position. The D genome of *Ae. tauschii* was already regarded by early cytogenetic studies as a rather well-separated lineage [43]. Some DNA marker-based studies placed it at basal position in the *Aegilops/Triticum* group [44-46].

According to our model, the most recent ancestor (MRCA) of the S genomes probably gained the group 5 gliadins. *Ae. searsii (S*^*s*^*), Ae. bicornis (S*^*b)*^*, Ae. sharonensis (S*^*sh*^*)* and *Ae. longissima (S*^*l*^*)* all have sequences of group 5 but none of group 6. *Ae. speltoides* (S) has group 6 sequences but none of group 5, in correspondence with it being the most divergent of the species of section Sitopsis [46-53]. Note that Eig [37] put *Ae. speltoides* in a separate subsection, *Truncata*, on the basis of morphological evidence. As the S genome species together are well separated from all other *Aegilops* species, they were by some considered as more closely related to *Triticum* than to other *Aegilops* species [54,55].

The species *Ae. caudata* (C), *Ae. umbellulata* (U), *Ae. comosa* (M) and *Ae. uniaristata* (N) share a common node in our model, representing a hypothetical common ancester that was differentiated from all other genomes by the combination of pseudogenes in group 1 gamma-gliadins and the absence of group 2 gamma-gliadins. From this ancestor the N and M genomes maintained group 4 gliadins, while the C and U genomes lost them. The similarity of *Ae. caudata* to *Ae. umbellulata* and *Ae. comosa* to *Ae. uniaristata* was already proposed by Kihara [43] and Lucas and Jahier [56] based on cytogenetic analysis, and by Dvorak and Zhang [48] based on RFLP data. A recent phylogenetic analysis of chloroplast haplotypes also showed similarity between the genomes of *Ae. comosa, Ae. uniaristata* and *Ae. caudata*[57].

### Evolution and selection of gamma-gliadins

A high level of genetic diversity was observed among gamma-gliadins, similarly to results of [3,10] and [14]. The number of groups in each genome reflects a more complicated evolution, over a longer period of time, than e.g. the alpha-gliadins of locus *Gli-2* on chromosome 6, which have been suggested to originate from a gliadin locus on chromosome 1 through a translocation event [5]. At the same time they do contain fewer pseudogenes that the 90% of alpha-gliadins [4]. The codon-based test for selection (Z-test) showed evidence for purifying selection in all groups of gamma-gliadin sequences (Table 2, Additional file 5) and at higher levels in intact genes than in pseudogenes. What mechanism made the gamma-gliadins split into separate groups, why is purifying selection stronger, and why do they have relatively few pseudogenes? One clue may come from the fact that the strength of selection, the variation in sequence length and in the number of cysteines, and the percentage pseudogenes, are clearly different between the six groups (Figure 3). This is most readily understood by comparing the most conserved and most polymorphic groups.

The most polymorphic is group 1, in which the genes encode proteins with 8 cysteines, which would allow them to be present as monomers. Deduced full sequences of this group varied in length from 762 (an *Ae. searsii* sequence from Genbank) to 1089 bp, which means that this group contains some of the shortest and all of the longest variants of the whole study. They were also most polymorphic in terms of sequence divergence, and the group is lost in many lineages (only maintained in *Lophopyrum*, *Crithiopsis*, *Dasypyrum*, and D and various S genomes) or consists of pseudogenes only (U and M genome). This suggests that as far as group 1 proteins perform any biological function, they are interchangeable with gliadins from other groups.

The most conserved are the group 6 gamma-gliadins, present in almost all *Aegilops* genome types (T, D, U, C, N, M and S (only *Ae. speltoides*)) and in *Lophopyrum*. They all have an uneven number of nine cysteines. The uneven number of cysteines would allow these proteins to become linked to a gluten network and function as a chain terminator. This particular group of gliadins is very conserved in length (all are 302 amino acids), except in *Lophopyrum*, where the additional cysteine is not present. The *Aegilops* species that do not have group 6 gamma-gliadins are the S genome species (except *Ae. speltoides*), all of which have group 5 gamma-gliadins, which are distinct in sequence composition but have the same length as the group 6 gliadins and have an additional cysteine in the same position. As a result, each *Aegilops* species has a group of 9-cysteine gamma-gliadins of a specific and conserved length. This strongly suggests that these 302 amino acid, 9-cysteine gamma-gliadins perform a specific function, possibly in relation to the gluten network formation during protein body formation in developing wheat grains. The traditional idea that gamma-gliadins have no free cysteines, and that all four S-S linkages (corresponding to 8 cysteines) are intramolecular, thus preventing gliadins from participating in the polymeric structure of glutenin, is clearly too simple. Altenbach et al. [58] already found several of these odd-numbered gamma-gliadins, but not yet in all genomes. The cysteines may be functional in combination with a fixed length if that provides a particular secondary structure (beta-reverse turns [59], possibly also related their capability to function as chain terminators in the polymer network).

Upelniek et al. [60] showed that differences in gliadin allele composition of Gli-1 loci among bread wheat varieties were correlated with differences in proteolysis rates during germination. Nevertheless, and apparently in contrast to the notion of specific functionality of at least some gamma-gliadins, hexaploid wheat appears to tolerate the loss of most or all gamma-gliadin proteins, as spring wheat cultivar Bobwhite grains remained viable when gamma-gliadin gene expression was mostly eliminated with RNAi [61] or when the bulk of all gliadins was silenced using an RNAi construct based on a conserved region from alpha-, gamma- and omega-gliadins [62]. However, Gil-Humanes et al. [63] did observe irregularities in the development of protein bodies in the endosperm when all gliadins were down-regulated, not only the gamma-gliadins. The effect of a reduction of gamma-gliadins by RNAi in commercial cultivars [64,65] or as a result of deletions in ‘Chinese Spring’ [66] is an increase in dough strength, which is consistent with a chain termination activity of part of the gamma-gliadins.

## Conclusion

We have studied the evolution of gamma-gliadins in diploid species of *Aegilops/Triticum* representing all main genome types in the group. Wide sampling enabled us to show that gamma-gliadins are represented by six diverged groups of genes that occur in different combinations across the genomes. The current gamma-gliadin composition in each of the genomes is the result of multiple gene duplication and divergence events followed by pseudogenisation within groups as well as loss of groups of genes during genome evolution. We have presented a possible model for duplications and deletions of groups of genes that proposes that at least some duplications predate the most recent common ancestor of all *Aegilops/Triticum* genomes that currently exist. Although the length and repeat composition are variable among genes, one specific type, a nine cysteine-containing gamma-gliadin of 302 amino acids, occurs in all *Aegilops* genomes, and these proteins may have a function in protein network formation.

## Competing interest

The authors declare that they have no competing interest.

## Authors’ contributions

SVG and MJMS initiated the study, NNC and EZA collected the material, SVG and EMJS designed the conserved primers and cloned the gamma-gliadins, SVG, EMJS and MJMS analysed the data, SVG, LJWJG, IMvdM, and MJMS wrote the paper. All authors read and approved the final manuscript.

## Supplementary Material

Additional file 1 List of all contigs, number of sequences, and Genbank accessions numbers. Click here for file

Additional file 2 Alignment of gamma-gliadin nucleotide sequences (Nexus format). Click here for file

Additional file 3 Alignment of gamma-gliadin amino acid sequences (Nexus format). Click here for file

Additional file 4 **Maximum-likelihood tree of the gamma-gliadins (based on nucleotide sequences) from diploid species of tribe Triticeae.** A maximum-likelihood (ML) analysis was performed with PhyML 3.0 using the GTR-substitution model. SH-like approximate likelihood-ratio test was used for estimation of branch support. Sequences that had length in the alignment less than 600 bp were excluded from the analysis. The gamma-gliadins fall into six groups (1–6 on the right) in two branches (1–2 and 3-4-5-6). Key for the sequence codes in Additional file 1. Click here for file

Additional file 5 **Ks/Ka ratio of intact and pseudogene gamma-gliadins.** Scatter plot of the numbers of synonymous substitutions (Ks) and non-synonymous substitutions (Ka) per site for pairwise comparisons among full open reading frame gamma-gliadins and pseudogene sequences. Linear trendlines with the intercept set to zero are shown both for full-open reading frame (ORF) sequences and pseudogene sequences. Click here for file

## References

[B1] Harberd NP Bartels D Thompson RD Analysis of the gliadin multigene loci in bread wheat using nullisomic-tetrasomic lines Mol Gen Genet 1985 198 234 242 10.1007/BF00383001

[B2] Okita TW Cheesbrough V Reeves CD Evolution and heterogeneity of the α-/β-type and γ-type gliadin DNA sequences J Biol Chem 1985 260 8203 8213 2989281

[B3] Anderson OD Litts JC Greene FC The α-gliadin gene family. I. Characterization of ten new wheat α-gliadin genomic clones, evidence for limited sequence conservation of flanking DNA, and southern analysis of the gene family Theor Appl Genet 1997 95 50 58 10.1007/s001220050531

[B4] Van Herpen TWJM Goryunova SV Van der Schoot J Alpha-gliadin genes from the A, B, and D genomes of wheat contain different sets of celiac disease epitopes BMC Genom 2006 7 1 10.1186/1471-2164-7-1 PMC1368968 16403227

[B5] Shewry PR Tatham AS The prolamin storage proteins of cereal seeds: structure and evolution Biochem J 1990 267 1 12 2183790 10.1042/bj2670001 PMC1131235

[B6] Payne PI Holt LM Jackson EA Law CN Wheat storage proteins: their genetics and their potential for manipulation by plant breeding Phil Trans R Soc Lond B 1984 304 359 371 10.1098/rstb.1984.0031

[B7] Payne PI Jackson EA Holt LM Law CN Genetic linkage between endosperm storage protein genes on each of the short arms of chromosomes 1A and 1B in wheat Theor Appl Genet 1984 67 235 243 10.1007/BF00317044 24258554

[B8] Sabelli P Shewry PR Characterization and organization of gene families at the Gli-1 loci of bread and durum wheat by restriction fragment analysis Theor Appl Genet 1991 83 209 216 10.1007/BF00226253 24202360

[B9] Anderson OD Hsia CC Torres V The wheat γ-gliadin genes: characterization of ten new sequences and further understanding of γ-gliadin gene family structure Theor Appl Genet 2001 103 323 330 10.1007/s00122-001-0551-3

[B10] Qi PF Wei YM Ouellet T Chen Q Tan X Zheng YL The γ-gliadin multigene family in common wheat (Triticum aestivum) and its closely related species BMC Genom 2009 10 168 10.1186/1471-2164-10-168 PMC2685405 19383144

[B11] Petersen G Seberg O Yde M Berthelsen K Phylogenetic relationships of Triticum and Aegilops and evidence for the origin of the A, B, and D genomes of common wheat (Triticum aestivum) Mol Phylogenet Evol 2006 39 70 82 10.1016/j.ympev.2006.01.023 16504543

[B12] Kilian B Ozkan H Deusch O Effgen S Brandolini A Kohl J Martin W Salamini F Independent wheat B and G genome origins in outcrossing Aegilops progenitor haplotypes Mol Biol Evol 2007 24 217 227 17053048 10.1093/molbev/msl151

[B13] Shewry PR Tatham AS Disulphide bonds in wheat gluten proteins J Cereal Sci 1997 25 207 227 10.1006/jcrs.1996.0100

[B14] Wang S Shen X Ge P Li J Subburaj S Li X Zeller FJ Hsam SL Yan Y Molecular characterization and dynamic expression patterns of two types of γ-gliadin genes from Aegilops and Triticum species Theor Appl Genet 2012 125 1371 1384 10.1007/s00122-012-1917-4 22751951

[B15] Zhang Q Donga Y An X Wang A Zhang Y Li X Gao L Xi X He Z Yan Y Characterization of HMW glutenin subunits in common wheat and related species by matrix-assisted laser desorption/ionization time-of-flight mass spectrometry (MALDI-TOF-MS) J Cereal Sci 2008 47 252 261 10.1016/j.jcs.2007.04.013

[B16] Li XH Zhang YZ Gao LY Wang AL Ji KM He ZH Molecular cloning, heterologous expression, and phylogenetic analysis of a novel y-type HMW glutenin subunit gene from the G genome of Triticum timopheevi Genome 2007 50 1130 1140 10.1139/G07-089 18059540

[B17] Zhang MY Wang K Wang SL Li XH Zeller FJ Hsam SLK Yan YM Molecular cloning, function prediction and phylogenetic analysis of LMW glutenin subunit genes in Triticum timopheevii (Zhuk.) Plant Breed 2010 129 622 629 10.1111/j.1439-0523.2010.01768.x

[B18] Wang S Li X Wang K Wang X Li S Zhang Y Guo G Zeller FJ Hsam SLK Yan Y Phylogenetic analysis of C, M, N, and U genomes and their relationships with Triticum and other related genomes as revealed by LMW-GS genes at Glu-3 loci Genome 2011 54 273 284 10.1139/g10-119 21491971

[B19] Van Slageren MW Wild wheats: a monograph of Aegilops L. and Amblyopyrum (Jaub. & Spach) Eig (Poaceae) Wag Ag Un P 1994 7 513

[B20] Edwards K Johnstone C Thompson C A simple and rapid method for the preparation of plant genomic DNA for PCR analysis Nucleic Acids Res 1991 19 1349 10.1093/nar/19.6.1349 2030957 PMC333874

[B21] Dorokhov DB Klocke EA Rapid and economic technique for RAPD analysis of plant genomes Russ J Genet 1997 33 358 365

[B22] Tamura K Dudley J Nei M Kumar S MEGA4: Molecular Evolutionary Genetics Analysis (MEGA) software version 4.0 Mol Biol Evol 2007 24 1596 1599 10.1093/molbev/msm092 17488738

[B23] Dereeper A Guignon V Blanc G (12 co-authors): Phylogeny.fr: robust phylogenetic analysis for the non-specialist Nucleic Acids Res 2008 36 W465 W469 10.1093/nar/gkn180 18424797 PMC2447785

[B24] Dereeper A Audic S Claverie JM Blanc G BLAST-EXPLORER helps you building datasets for phylogenetic analysis BMC Evol Biol 2010 10 8 10.1186/1471-2148-10-8 20067610 PMC2821324

[B25] Anisimova M Gascuel O Approximate likelihood ratio test for branchs: A fast, accurate and powerful alternative Syst Biol 2006 55 539 52 10.1080/10635150600755453 16785212

[B26] Nei M Gojobori T Simple methods for estimating the numbers of synonymous and nonsynonymous nucleotide substitutions Mol Biol Evol 1986 3 418 426 3444411 10.1093/oxfordjournals.molbev.a040410

[B27] Salentijn EMJ Mitea DC Goryunova SV Van der Meer IM Padioleau I Gilissen LJWJ Koning F Smulders MJM Celiac disease T cell epitopes from gamma-gliadins: immunoreactivity depends on the genome of origin, transcript frequency, and flanking protein variation BMC Genomics 2012 13 277 10.1186/1471-2164-13-277 22726570 PMC3469346

[B28] Bowden WM The taxonomy and nomenclature of the wheats, barleys, and ryes and their wild relatives Can J Bot 1959 37 657 684 10.1139/b59-053

[B29] Kimber G Sears ER Heyne EG Evolution in the genusTriticumand the origin of cultivated wheat Wheat and Wheat Improvement 1987 2 Madison, WI: Am. Soc. Agron 154 164

[B30] Huang S Sirikhachornkit A Su X Faris J Gill B Haselkorn R Gornicki P Genes encoding plastid acetyl-CoA carboxylase and 3-phosphoglycerate kinase of the Triticum/Aegilops complex and the evolutionary history of polyploid wheat Proc Natl Acad Sci USA 2002 99 8133 8138 10.1073/pnas.072223799 12060759 PMC123033

[B31] Clegg MT Cummings MP Durbin ML The evolution of plant nuclear genes Proc Natl Acad Sci USA 1997 94 7791 7798 10.1073/pnas.94.15.7791 9223265 PMC33705

[B32] Pan Q Wendel J Fluhr R Divergent evolution of plant NBS-LRR resistance gene homologues in dicot and cereal genomes J Mol Evol 2000 50 203 213 10754062 10.1007/s002399910023

[B33] Gao S Gu YQ Wu J Coleman-Derr D Huo N Crossman C Jia J Zuo Q Ren Z Anderson OD Kong X Rapid evolution and complex structural organization in genomic regions harboring multiple prolamin genes in the polyploid wheat genome Plant Mol Biol 2007 65 189 203 10.1007/s11103-007-9208-1 17629796

[B34] Nei M Gu X Sitnikova T Evolution by the birth-and-death process in multigene families of the vertebrate immune system Proc Natl Acad Sci USA 1997 94 7799 7806 10.1073/pnas.94.15.7799 9223266 PMC33709

[B35] Escobar JS Scornavacca C Cenci A Guilhaumon C Santoni S Douzery EJP Ranwez V Glémin S David J Multigenic phylogeny and analysis of tree incongruences in Triticeae (Poaceae) BMC Evol Biol 2011 11 181 10.1186/1471-2148-11-181 21702931 PMC3142523

[B36] Zhukovsky PM A critical systematic survey of the species of the genus Aegilops L B Appl Botany, Genet Plant Breeding 1928 18 497 609

[B37] Eig A Monographisch-kritische Übersicht der Gattung Aegilops Feddes Repertorium Specierum novarum regni vegetabilis Beih 1929 55 1 228

[B38] Kihara H Fertility and morphological variation in the substitution backcrosses of the hybrid Triticum vulgare × Aegilops caudata Proc X Int Congr Genet 1959 1 142 171

[B39] Hammer K Vorarbeiten zur monographischen Darstellung von Wildpflanzensortimenten: Aegilops L Kulturpflanze 1980 28 33 180 10.1007/BF02014641

[B40] Whitcombe JR A guide to the species of Aegilops L.: their taxonomy, morphology, and distribution 1983 Rome, Italy: International Board for Plant Genetic Resources (IPGRI) 74

[B41] Eig A Amblyopyrum Eig. A new genus separated from the genus Aegilops PZE Ins Agr Nat Hist Agr Res 1929 2 199 204

[B42] Badaeva E Friebe B Gill B Genome differentiation in Aegilops. 1. Distribution of highly repetitive DNA sequences on chromosomes of diploid species Genome 1996 39 293 306 10.1139/g96-040 18469894

[B43] Kihara H Considerations on the evolution and distribution of Aegilops species based on the analyser-method Cytologia 1954 19 336 357 10.1508/cytologia.19.336

[B44] Dvorak J Zhang HB Reconstruction of the phylogeny of the genus Triticum from variation in repeated nucleotide sequences Theor Appl Genet 1992 84 419 429 10.1007/BF00229502 24203203

[B45] Dvorak J Luo M-C Yang Z-L Restriction fragment length polymorphism and divergence in the genomic regions of high and low recombination in self-fertilizing and cross-fertilizing Aegilops species Genetics 1998 148 423 434 9475752 10.1093/genetics/148.1.423 PMC1459766

[B46] Dvorak J Luo M-C Yang Z-L Zhang H-B The structure of the Aegilops tauschii genepool and the evolution of hexaploid wheat Theor Appl Genet 1998 97 657 670 10.1007/s001220050942

[B47] Ogihara Y Tsunewaki K Diversity and evolution of chloroplast DNA in Triticum and Aegilops as revealed by restriction fragment analysis Theor Appl Genet 1988 76 321 332 10.1007/BF00265331 24232195

[B48] Dvorak J Zhang HB Variation in repeated nucleotide sequences sheds light on the phylogeny of the wheat B and G genomes Proc Natl Acad Sci USA 1990 87 9640 9644 10.1073/pnas.87.24.9640 11607134 PMC55228

[B49] Miyashita NT Monri N Tsunewaki K Molecular variation in chloroplast DNA regions in ancestral species of wheat Genetics 1994 137 883 889 7916310 10.1093/genetics/137.3.883 PMC1206048

[B50] Sasanuma T Miyashita NT Tsunewaki K Wheat phylogeny determined by RFLP analysis of nuclear DNA. 3. Intra- and interspecific variations of five Aegilops Sitopsis species Theor Appl Genet 1996 92 928 934 10.1007/BF00224032 24166619

[B51] Dvorak J Luo M-C Yang Z-L Damania A Genetic evidence on the origin of T. aestivum L The origins of agriculture and the domestication of crop plants in the Near East 1998 Aleppo, Syria, ICARDA: ICARDA 235 251

[B52] Giorgi D D'Ovidio R Tanzarella OA Porceddu E RFLP analysis of Aegilops species belonging to the Sitopsis section Genet Resour Crop Evol 2002 49 145 151 10.1023/A:1014743823887

[B53] Goryunova SV Kochieva EZ Chikida NN Pukhalskyi VA Phylogenetic relationships and intraspecific variation of D-genome Aegilops L. as revealed by RAPD analysis Russ J Genet 2004 40 515 523 15272562

[B54] Chennaveeraiah MA Karyomorphologic and Cytotaxonomic Studies in Aegilops Acta Horti Gotoburgensis 1960 23 85 178

[B55] Zhukovskii PM Kul’turnye rasteniya i ikh sorodichi (Cultivated Plants and Their Relatives) 1971 Kolos, Leningrad 122 130 in Russian

[B56] Lucas H Jahier J Phylogenetic relationships in some diploid species of Triticineae: cytogenetic analysis of interspecific hybrids Theor Appl Genet 1988 75 498 502 10.1007/BF00276756

[B57] Meimberg H Rice KJ Milan NF Njoku CC Mckay JK Multiple origins promote the ecological amplitude of allopolyploid Aegilops (Poaceae) Am J Bot 2009 96 1262 1273 10.3732/ajb.0800345 21628275

[B58] Altenbach SB Vensel WH DuPont FM Analysis of expressed sequence tags from a single wheat cultivar facilitates interpretation of tandem mass spectrometry data and discrimination of gamma gliadin proteins that may play different functional roles in flour BMC Plant Biol 2010 10 7 10.1186/1471-2229-10-7 20064259 PMC2827424

[B59] Gianibelli MC Larroque OR MacRitchie F Wrigley CW Biochemical, genetic, and molecular characterization of wheat endosperm proteins Online review 2001 St. Paul, Minnesota, USA: American Association of Cereal Chemists, Inc 1 20 Publication no. C-2001-0926-01O

[B60] Upelniek VP Brezhneva TA Dadashev SY Novozhilova OA Molkanova OI Semikhov VF On the use of alleles of gliadin-coding loci as possible adaptability markers in the spring wheat (Triticum aestivum L.) cultivars during seed germination Russ J Genet 2003 39 1426 1431 14964836

[B61] Gil-Humanes J Pistón F Hernando A Alvarez JB Shewry PR Barro F Silencing of γ-gliadins by RNA interference (RNAi) in bread wheat J Cereal Sci 2008 48 565 568 10.1016/j.jcs.2008.03.005

[B62] Gil-Humanes J Pistón F Tollefsen S Sollid LM Barro F Effective shutdown in the expression of celiac disease-related wheat gliadin T-cell epitopes by RNA interference Proc Natl Acad Sci USA 2010 107 17023 17028 10.1073/pnas.1007773107 20829492 PMC2947919

[B63] Gil-Humanes J Pistón F Shewry PR Tosi P Barro F Suppression of gliadins results in altered protein body morphology in wheat J Exp Bot 2011 62 4203 4213 10.1093/jxb/err119 21561951

[B64] Pistón F Gil-Humanes J Rodríguez-Quijano M Barro F Down-regulating γ-gliadins in bread wheat leads to non-specific increases in other gluten proteins and has no major effect on dough gluten strength PLoS ONE 2011 6 e24754 10.1371/journal.pone.0024754 21935456 PMC3172295

[B65] Gil-Humanes J Pistón F Giménez MJ Martín A Barro F The Introgression of RNAi Silencing of γ-Gliadins into Commercial Lines of Bread Wheat Changes the Mixing and Technological Properties of the Dough PLoS ONE 2012 7 e45937 10.1371/journal.pone.0045937 23029328 PMC3454332

[B66] Van den Broeck HC Van Herpen TWJM Schuit C Salentijn EMJ Dekking L Bosch D Hamer RJ Smulders MJM Gilissen LJWJ Van der Meer IM IM Removing celiac disease-related gluten proteins from bread wheat while retaining technological properties: a study with Chinese Spring deletion lines BMC Plant Biol 2009 9 41 10.1186/1471-2229-9-41 19351412 PMC2670835

