# Supplementary figures and images for: Expansion of the gamma-gliadin gene family in Aegilops and Triticum
Source: BMC Evol Biol. 2012 Nov 8;12:215. doi: 10.1186/1471-2148-12-215 (PMC3537741; doi:10.1186/1471-2148-12-215)

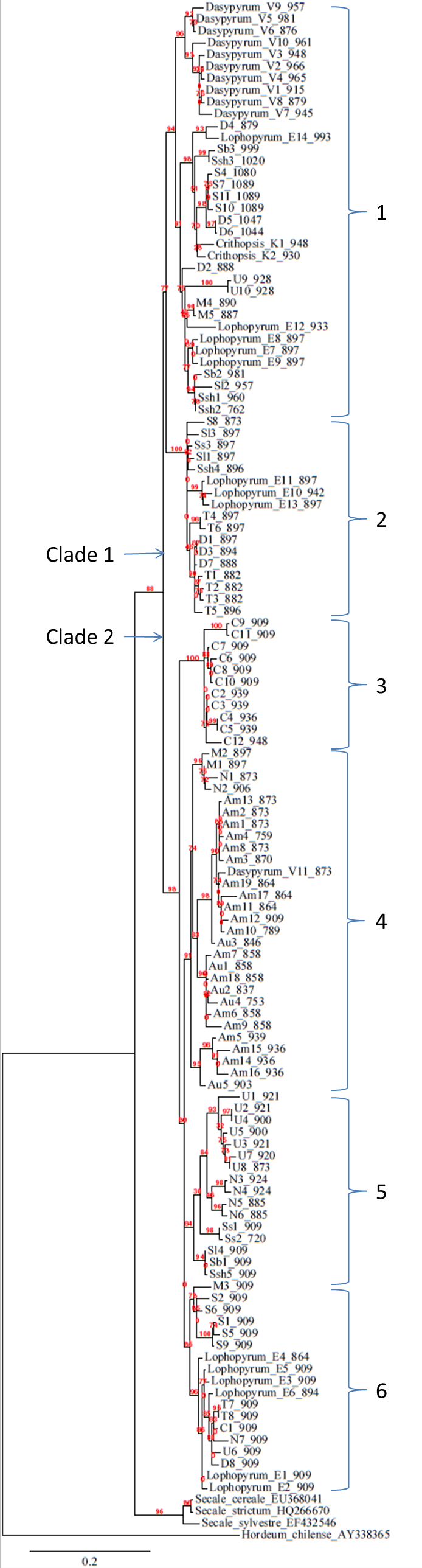

Supplement: Additional file 4 — Maximum-likelihood tree of the gamma-gliadins (based on nucleotide sequences) from diploid species of tribe Triticeae. A maximum-likelihood (ML) analysis was performed with PhyML 3.0 using the GTR-substitution model. SH-like approximate likelihood-ratio test was used for estimation of branch support. Sequences that had length in the alignment less than 600 bp were excluded from the analysis. The gamma-gliadins fall into six groups (1–6 on the right) in two branches (1–2 and 3-4-5-6). Key for the sequence codes in Additional file 1. [file 1471-2148-12-215-S4.tiff]
