# Supplementary material for: Expansion of the gamma-gliadin gene family in Aegilops and Triticum
Source: BMC Evol Biol. 2012 Nov 8;12:215. doi: 10.1186/1471-2148-12-215 (PMC3537741; doi:10.1186/1471-2148-12-215)
Supplement: Supplementary file 5 [file 1471-2148-12-215-S5.docx]

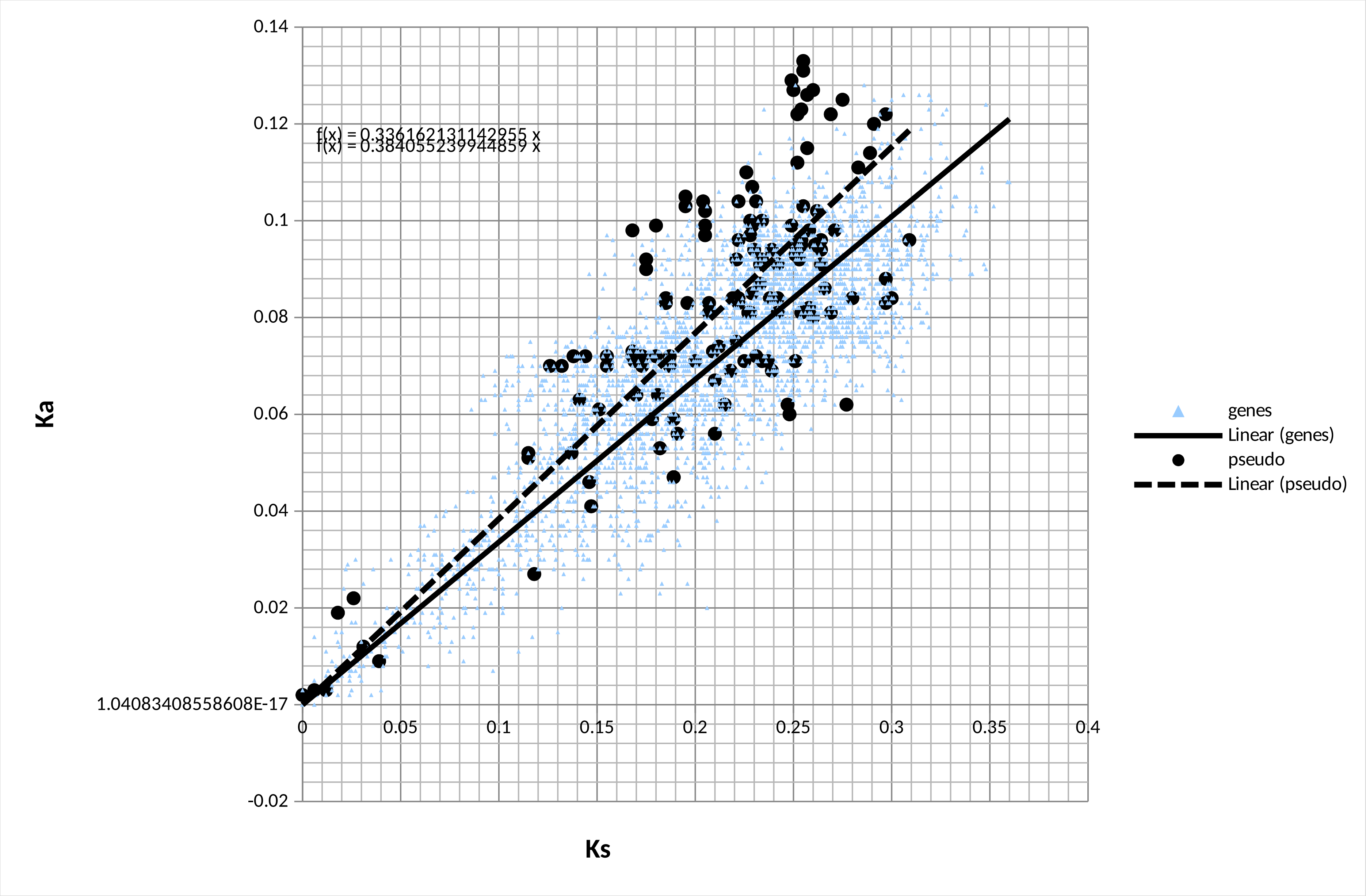
0.14
0.12
0.1
0.08
0.06
0.04
0.02
1.04083408558608E-17
-0.02
0
0.05
0.1
0.15
0.2
0.25
0.3
0.35
0.4
f(x) = 0.336162131142955 x
f(x) = 0.384055239944859 x
Ka
Ks
genes
Linear (genes)
pseudo
Linear (pseudo)
